# An Approach of Producing Ultra-High-Performance Concrete with High Elastic Modulus by Nano-Al_2_O_3_: A Preliminary Study

**DOI:** 10.3390/ma15228118

**Published:** 2022-11-16

**Authors:** Hongyan Chu, Qun Wang, Li Gao, Jinyang Jiang, Fengjuan Wang

**Affiliations:** 1 College of Civil Engineering, Nanjing Forestry University, Nanjing 210037, China; 2 School of Materials Science and Engineering, Southeast University, Nanjing 211189, China

**Keywords:** ultra-high-performance concrete, mechanical properties, microstructure, porosity, nano alumina, drying shrinkage, elastic modulus

## Abstract

Ultra-high-performance concrete (UHPC) has promising applications in civil engineering. However, the elastic modulus of UHPC is relatively low compared with its compressive strength, which may result in insufficient stiffness in service. This work was carried out to explore the feasibility of producing UHPC with high elastic modulus by nano-Al_2_O_3_ (NA). Based on particle densely packing theory, the initial mixture of UHPC was designed via the modified Andreasen and Andersen model. An experimental investigation was conducted to systematically examine the effects of NA on different properties of UHPC, including its fluidity, mechanical properties, durability, and microstructure. It was found that: (1) Compared with UHPC without NA, the flexural strength, compressive strength, and elastic modulus of UHPC were improved by 7.38–16.87%, 4.08–20.58%, and 2.89–14.08%, respectively, because of the incorporation of NA; (2) the addition of NA had a prohibiting impact on the threshold pore diameter and porosity of UHPC, which suggested that NA could be conducive to its pore structure; (3) the incorporation of NA led to a decline of 2.9–11.76% in the dry shrinkage of UHPC, which suggested that incorporating NA in a proper amount could reduce the risk of cracking and alleviate the dry shrinkage of UHPC; (4) the optimal amount of NA in UHPC was 1.0%, considering the effects of NA on workability, mechanical properties, microstructure, and the durability of UHPC.

## 1. Introduction

Ultra-high-performance concrete (UHPC) is a kind of concrete that has been verified to perform well in durability and mechanical properties. Its compressive strength is usually above 120 MPa, and the elastic modulus reaches 40–60 GPa [1]. UHPC meets the needs of sustainable development and is currently a popular research topic in civil engineering [2]. Currently, UHPC is mainly used in bridge engineering, aquatic structure, construction, and municipal engineering [3,4]. In the domain of structural design, the elastic modulus is a significant parameter of concrete and one of the important elements of UHPC research. At present, UHPC is roughly 1 time and 2–3 times higher, respectively, than high-performance and ordinary concrete in terms of compressive strength but merely 1/2 times greater than ordinary and high-performance concrete in terms of elastic modulus. The elastic modulus of UHPC does not increase in the same magnitude as its compressive strength, which can lead to insufficient stiffness and excessive deformation of the UHPC structure, while its structural strength is acceptable [1]. In contrast, increasing the elastic modulus is a way to improve the overall stiffness of concrete structures and alleviate the deformation of concrete structures under load to a certain extent. Therefore, to expand the application of UHPC in engineering, it is extremely important to effectively improve its elastic modulus.

Compared with ordinary concrete, many raw materials and fibers are usually used in UHPC, so its elastic modulus is affected by a large number of factors. The current research on UHPC and its elastic modulus, both at home and abroad, is focused on supplementary cementitious materials, coarse aggregates, etc. Hannawi et al. [5] studied how UHPC is influenced by various fibers with regard to its elastic modulus and found that steel fibers perform better than other types of fibers in improving UHPC from the perspective of elastic modulus. UHPC modified by graphene oxide was found in a study conducted by Chu et al. [2] to have a higher elastic modulus than the reference group. At the same time, domestic and foreign scholars also found that UHPC’s elastic modulus is significantly affected by the coarse aggregate in dimensions including particle size range, contents, and elastic modulus. UHPC’s elastic modulus, as revealed by Piasta et al. [6], was significantly promoted through the use of basalt aggregate with high strength and high rigidity. UHPC added with granite and basalt aggregate was found by Wu et al. [7] to have a greater elastic modulus than UHPC with river sand. UHPC with coarse basalt aggregate was observed by Ouyang et al. [8] to reach more than 55 GPa in elastic modulus.

Nanomaterials can improve the mechanical properties and durability performance of cement-based materials due to their promoting effect on their microstructure and are therefore widely used in cement-based materials [9,10]. More and more types of nanomaterials arising from the development of nanotechnology have been incorporated into cementitious materials, such as nano-SiO_2_ [11], nano-Al_2_O_3_ (NA) [12], graphene oxide [13], nano-CaCO_3_ [14], and nano-TiO_2_ [15]. Compared with other nanomaterials, NA has a higher specific surface area. The main chemical composition of NA is Al_2_O_3_, which has high activity in the process of the cement hydration reaction [16]. Therefore, the incorporation of NA into concrete may improve its mechanical properties and durability. Feng et al. [17] found that the incorporation of NA improved the mechanical properties and workability of magnesium phosphate cement composite. Heika et al. [18] found that cement paste added with NA at a proportion of 1% had the maximum compressive strength. Bahareh et al. [19] found that self-compacting mortar added with NA had significant improvement in its durability and some improvement in its compressive strength and flexural strength. Joshaghani et al. [20] found that self-compacting concrete could have significant improvement in its durability and a certain degree of enhancement in its mechanical properties through the incorporation of NA in a small amount. The addition of alumina nanoparticles, as pointed out by Meddah et al. [21], could improve the strength and durability of concrete. In addition, some ceramics with Al_2_O_3_ could be used as a shielding material or electrochemical devices in nuclear technology [22,23]. Nonetheless, little attention has been paid to whether UHPC added with NA could have higher elastic modulus. As for the incorporation, the focus of most studies is placed on its impact on cement paste or ordinary concrete with regard to mechanical properties or durability. In view of this research gap, this study was conducted to explore the changes in UHPC added with NA in workability and mechanical properties.

The mix design, one of the important aspects determining the production and application of concrete, directly affects all parameters of UHPC. Regarding the mix design of UHPC, the compressible model (CPM) [24,25] and the Fuller [26] model are the most typical models. Based on the Fuller model, Funk and Dinger put forward the modified Andreasen and Andersen (MAA) particle-packing model, which is a classical continuous particle-packing model widely used in concrete mix design due to its use of a non-single particle size distribution for stacking [27,28,29]. Yang et al. [30] studied the effect of this model in optimizing the gradation of materials and used quartz chips to replace quartz sand in a kind of green UHPC. In accordance with this model, Li et al. [31] rendered the largest particle size of 16 mm in the use of basalt coarse aggregate to design UHPC. This model was also followed by Wang et al. [32], who replaced the cement and aggregate in UHPC with construction demolition waste, leading to the creation of a green UHPC that met sustainable development.

The MAA particle-packing model, by virtue of its advantages and NA’s excellent performance as a supplementary cementitious material, was applied in this work for the initial mix design of UHPC, whereby UHPC’s elastic modulus was hoisted by the incorporation of NA. On this basis, the effects of different amounts of NA on a number of parameters of UHPC including its durability, mechanical properties, and workability were investigated. Moreover, techniques such as scanning electron microscopy and mercury intrusion porosimetry were applied to investigate the changes in UHPC in microstructure under the action of NA. The present work is instructive for ensuring a high elastic modulus in the preparation of UHPC using NA. In addition, the UHPC with high elastic modulus is expected to be used in civil engineering, construction engineering, hydraulic engineering, tunnel engineering, bridge engineering, nuclear power engineering, and security engineering.

## 2. Experiment

### 2.1. Experimental Materials

**Figure 1:**
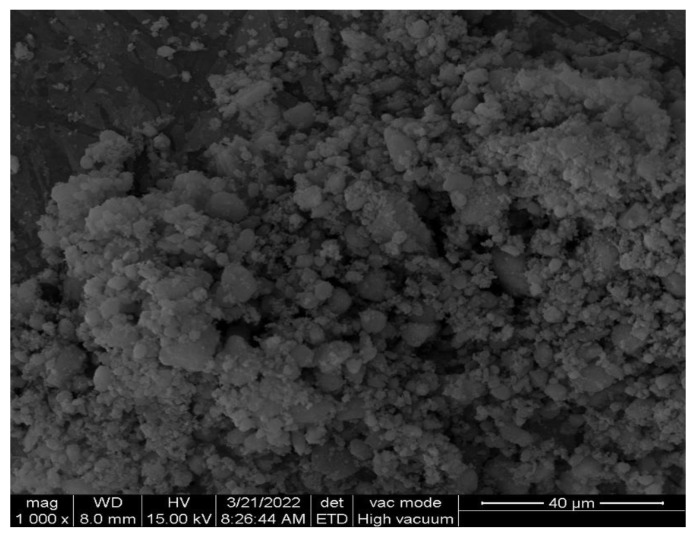
SEM image of NA.

The materials selected for use in this work included: (1) P∙O 52.5 grade cement from Yangchun Shanshui Cement Co., Ltd. (Weifang, China)with a loss on ignition of 1.4%; (2) class I fly ash produced by Yuanheng Water Purification Material Processing Plant (Gongyi, China); (3) silica fume of 940 grade produced by Elkem International Trading Co., Ltd. (Shanghai, China); (4) quartz sand from Taizhou Qunxiao New Material Co., Ltd. (Taizhou, China), which was mixed in accordance with the mass ratio of 1:1:1 by three different particle sizes of 0.5–1, 1–2 and 2–4 mm; (5) NA produced by Yun Guan Biotechnology Co., Ltd. (Shanghai, China), of which the microstructure of NA is shown in Figure 1. The environmental scanning electron microscope FEI Quanta 200 (Hillsboro, OR, USA)was employed to analyze the microstructure of NA. Gold spraying of the surface of NA sample was finished before scanning. The particle size diameter of NA was 10 nm, and the purity of NA was 99.99%; (6) high-efficiency polycarboxylic acid water-reducing agent, with a water-reducing rate above 33% and a solid content of 40%, from Jiangsu Subute New Material Co., Ltd. (Nanjing, China); and (7) tap water. The chemical compositions of the four types of materials used in the experiment are shown in Table 1, which were determined through X-ray fluorescence.

### 2.2. Mix Design of UHPC

The tight packing between the particles of the experimental material plays a decisive role in obtaining a dense structure of that material [33]. As shown in Equation (1), the minimum porosity of the mixture could be theoretically obtained by the optimum particle size distribution (PSD) of each kind of particulate material used in the mixture:

(1) $$P\left(D\right)={\left(\frac{D}{D_{\max}}\right)}^{q}$$

In Equation (1), P(D) stands for the fraction of the total solids with a size less than D; q represents the distribution modulus; D is the particle size (μm); and D_max_ is the maximum particle size. Equation (1) does not take the minimum particle size into account, and the particle size must have a finite lower limit. The MAA particle-packing model developed upon the Andreasen and Andersen [34] equation by Funk and Dinger is shown in Equation (2):

(2) $$P\left(D\right)=\frac{\begin{array}{ccc} D_{i}^{q} & - & D_{\min}^{q} \end{array}}{\begin{array}{ccc} D_{\max}^{q} & - & D_{\min}^{q} \end{array}}$$

In Equation (2), D_min_ is the minimum particle size.

The ratio of coarse particles to fine particles is determined by q, whereby a higher distribution modulus (q > 0.5) means that the particles in the mixture are coarser, while the lower the q, the finer particles in the mixture [30]. Therefore, q was taken as 0.23 herein, considering the higher number of fine particles used in UHPC in this study [35].

**Figure 2:**
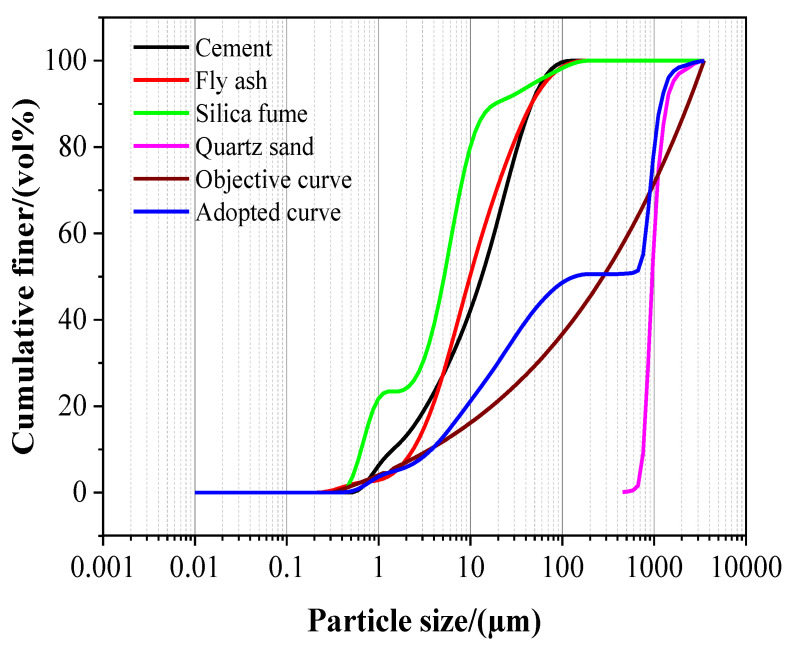
Particle size distribution of the raw materials.

Based on the above-mentioned particle densely packing theory, the best distribution position between the granules of the mixed material and the best fit between the target curve and the mixture was calculated. The results of the calculation are shown in Figure 2.

As shown in Table 2, all the mixtures used in this study had a steel fiber content of 2% (volume fraction) and a water–cement ratio of 0.185. NA0 represents UHPC with 0% NA (control group); NA0.5 represents UHPC with 0.5% NA; NA1.0 represents UHPC with 1.0% NA; NA1.5 represents UHPC with 1.5% NA; NA2.0 represents UHPC with 2.0% NA (mass of cement).

### 2.3. Specimen Preparation and Curing

The following steps were involved in the production of the five types of UHPC: (1) An ordinary mixer was added with the required cement, fly ash, silica fume, and NA, and the mixture was stirred for four minutes in drying condition; (2) then the mixture was added with the weighed quartz sand and stirred for three minutes in drying condition; (3) the mixture was added with three-fourths of the water, stirred with a glass rod after the selected water-reducing agent was added, and stirred for three minutes; (4) the mixture was added with the fluid from the glass rod and the containers of water-reducing agent rinsed with the rest water and stirred for five minutes; (5) finally, the mixture was evenly added with the weighed steel fibers and stirred for five minutes.

As shown in Table 2, a number of UHPC specimens in difference sizes such as 40 × 40 × 160 mm^3^, 25 × 25 × 280 mm^3^, and 100 × 100 × 300 mm^3^ were prepared in accordance with the respective mix proportions. After the completion of preparation, the specimens were cured naturally for 24 h in the mixing room. In the next step, they were taken off the molds and cured till the corresponding date for testing in a standardized curing environment where the relative humidity was 95% or above and the temperature was between 18 and 22 °C. The specimens were cured until the corresponding testing age.

### 2.4. Experimental Methods

Chinese national standard GB/T2419-2005 [36] was referred to in the process of conducting the test of fluidity. A truncated cone mold in a size of 70 × 100 × 60 mm^3^ was added with the freshly made mixture of UHPC twice. A tamping rod was used to tamp the mixture in the mold. Eventually, the filled mold was made to jump for 25 times on the jumping table. The diameters on the bottom surface, which are perpendicular to each other, were measured with a ruler. The mean of the measured results was used to represent the value of fluidity.

The Chinese National Standard GB/T 17671-1999 [37] was referred to in the process of measuring the mechanical properties of UHPC, including its flexural strength and compressive strength, to examine the changes in these parameters after the addition of NA into UHPC. Three specimens in each group, in the size of 40 × 40 × 160 mm^3^, were selected for the following test and cured in a standardize condition for 28 days. Three experimental measurements were calculated to acquire the means, which were used to represent UHPC at the curing age in the two parameters tested.

At a loading rate of 1 MPa/s, universal testing machine, resistance strain gauge, and TDS-530 acquisition instrument were utilized to measure UHPC in terms of elastic modulus. The specimens in a size of 100 × 100 × 300 mm^3^ were selected for testing and cured for 28 days. The Chinese National Standard GB/T 50081-2019 [38] was referred to in the process of measuring the elastic modulus of UHPC. The test was repeated six times to obtain the mean UHPC in elastic modulus. At the testing load of 30–60% of the axial compressive strength, it was found that there was favorable linearity with regard to stress and strain. Herein, UHPC in the elastic modulus was determined to be the secant modulus on the stress–strain curve at 40% of its stress peak [39].

The technical specification CECS 02:2005 [40] was referred to in the process of testing each group of UHPC specimens with BJNM-1 nonmetallic ultrasonic detector. There were three tested specimens at the curing age of 28 days in each of the five groups, each in the size of 100 × 100 × 300 mm^3^. The test was repeated three times to obtain the mean of UHPC in ultrasonic pulse velocity.

After the hardening of the UHPC specimens, their changes in length were tested using the BC156-300 length comparator with the accuracy rate of 0.001 mm from Hebei Yuhengyuan Instrument Trading Co., Ltd. UHPC specimens in the size of 25 × 25 × 285 mm^3^ were used to test drying shrinkage. L_0_ (initial length of the specimens) was measured after demolding and drying the surface of each specimen through wiping. Afterwards, they were cured for 1, 3, 7, 14, 28, and 56 days in a standardized environment in which relative humidity was between 55% and 65% and the temperature was between 18 and 22 °C. The length of each specimen at different curing ages was measured. Moreover, Equation (3) was followed to calculate the drying shrinkage of UHPC:

(3) $$\varepsilon =\frac{L_{0}-L_{t}}{L-L_{d}}$$

In Equation (3), ε refers to the drying shrinkage; L_0_ stands for the initial length; L is 285 mm, standing for the preparation length; L_t_ represents the length at a specific curing age; and L_d_ is 20 ± 2 mm, referring to the length of the copper head segment that was embedded into UHPC specimens.

Mercury intrusion porosimetry, namely Micrometrics Autopore IV9500 (Norcross, GA, USA), was applied to measure the parameters of UHPC, including its pore size distribution and porosity, for the purpose of examining the effect of NA on the microstructure of UHPC. The samples of UHPC made to measure mechanical properties were cut to get the samples for this measurement. The samples were used for testing after hydration was terminated in absolute ethanol and after they were dried at 60 °C for seven days in a vacuum drying oven. There was a total of nine samples, cured for 28 days, used for testing. The test was repeated three times for each kind of UHPC.

Environmental scanning electron microscope FEI Quanta 200 was employed to analyze how the microstructure of UHPC changed under the action of NA. Gold spraying of the surface of each UHPC sample was finished before scanning. The samples of UHPC made to measure mechanical properties were cut to acquire the samples for this measurement, with 28 days’ curing age conducted on each experimental sample.

## 3. Results and Discussion

### 3.1. Fluidity

**Figure 3:**
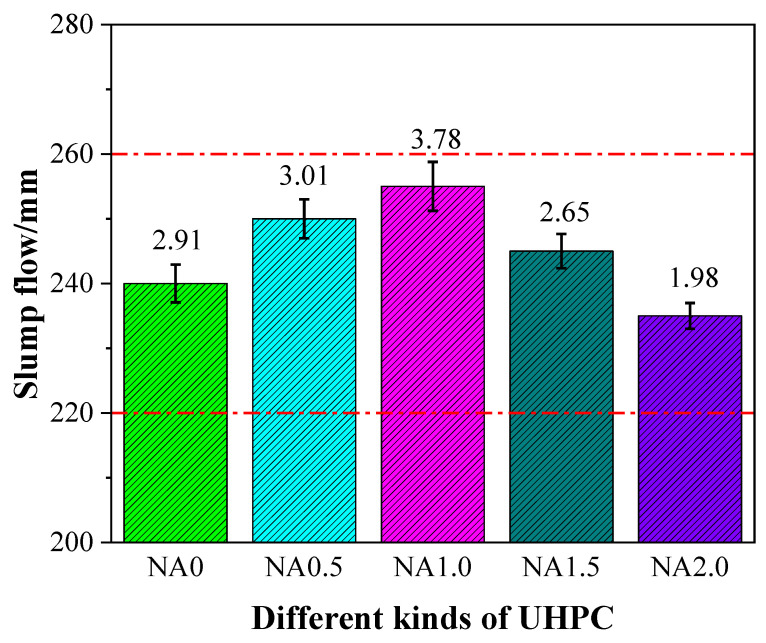
Slump flow of UHPC with different contents of NA.

The slump flow of UHPC had some changes, as shown in Figure 3, due to the incorporation of NA in varying proportions. To be specific, it experienced a rising tendency and then underwent a declining tendency. The slump flow was 240, 250, 255, 245, and 235 mm for the examined types of UHPC ranging from NA0 to NA2.0. These values of slump flow within 240 ± 20 mm, a ranged required for self-compacting mortar, hinted at the qualification of UHPC to work as self-compacting mortar in all cases [41]. When the content of NA was not greater than 1.0%, the increasing proportion of NA led to the rise in the slump flow of UHPC. When greater than 1.0%, the increasing proportion of NA led to the decline in the slump flow of UHPC. The slump flow of UHPC added with NA at the ratio of 1.0% was better at 255 mm, remarkably, 6.25% higher than the control group. UHPC added with NA at the ratio of 1.0% was found to have the highest slump flow. These results showed that while fluidity was used as the explained variable, 1.0% is the most optimum proportion of NA in UHPC. There may be the following reasons accounting for the changes of UHPC in slump flow with the rising proportion of NA: (1) the particle size of NA was 10 nm, which could fill the void between the raw materials inside UHPC, so that the number of voids and the pore size inside UHPC could be reduced. Thus, the filling of these voids and pores consumed less water, and the remaining water improved the fluidity of UHPC [42]; (2) with the increase in NA, it might agglomerate within the UHPC, resulting in the poor dispersion of NA in UHPC and thereby leading to the decline in its slump flow [43].

### 3.2. Flexural Strength

**Figure 4:**
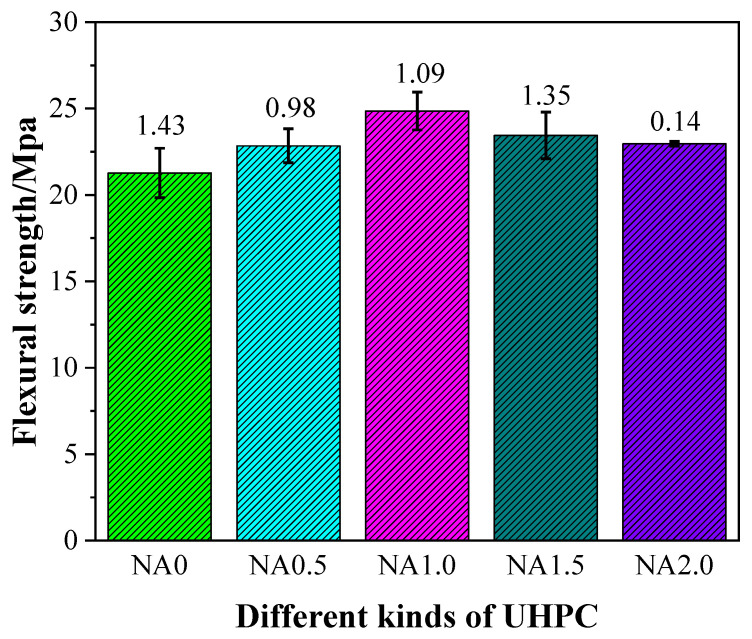
Flexural strength of UHPC with different contents of NA.

The flexural strength of UHPC had some changes, as shown in Figure 4, due to the incorporation of NA at varying proportions. Specifically, UHPC with NA was found to have a higher level of flexural strength than UHPC without NA, hinting at the promoting effect of the addition of NA on the flexural strength. The flexural strength at the curing age of 28 days was 21.27, 22.84, 24.86, 23.44, and 22.96 MPa for the examined types of UHPC ranging from NA0 to NA2.0. The flexural strengths of NA0.5, NA1.0, NA1.5, and NA2.0 at the curing age of 28 days were, respectively, 7.38%, 16.87%, 10.20%, and 7.94% higher than that of NA0. UHPC added with NA at the ratio of 1.0% was observed to have the highest flexural strength. Therefore, when flexural strength was treated as the explained variable, UHPC performed best when the proportion of NA was 1.0%.

The flexural strength of UHPC added with NA in this work at varying ratios at the curing age of 28 days was somewhat greater than that of UHPC in the work by Mo et al. [44], generally within 21.27–24.86 MPa. The addition of NA triggered the pozzolanic reaction, which was the major cause of the increased flexural strength of UHPC because it improved the interfacial transition zone of the UHPC matrix, eventually improving the fiber–matrix adhesion [45].

### 3.3. Compressive Strength

**Figure 5:**
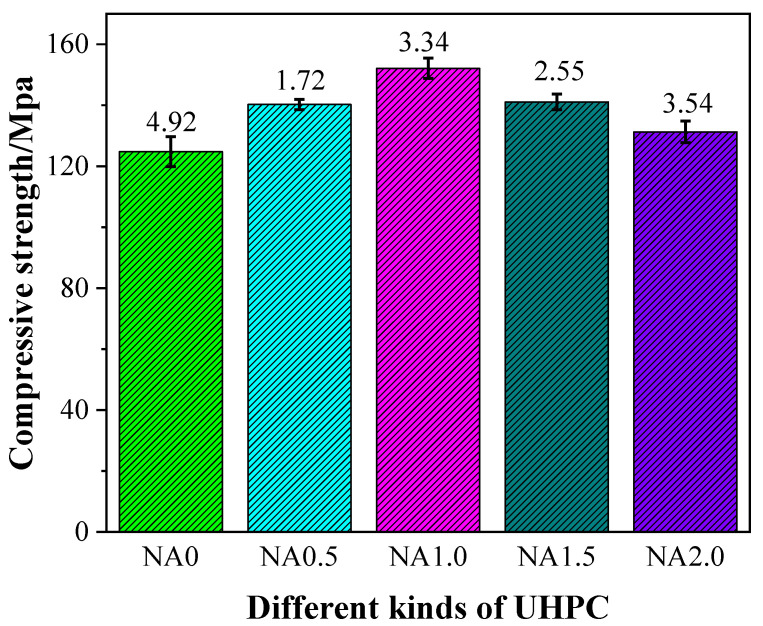
Compressive strength of UHPC with different contents of NA.

The compressive strength of UHPC had some changes, as shown in Figure 5, due to the incorporation of NA at varying proportions. Specifically, UHPC with NA was found to have a higher level of compressive strength than UHPC without NA, hinting at the promoting effect of the addition of NA on the compressive properties of UHPC. The compressive strengths at the curing age of 28 days were 126.11, 140.25, 152.06, 141.08, and 131.25 MPa for the examined types of UHPC ranging from NA0 to NA2.0. The compressive strengths of NA0.5, NA1.0, NA1.5, and NA2.0 at the curing age of 28 days were, respectively, 11.21%, 20.58%, 11.87%, and 4.08% higher than that of NA0, indicating the nonlinear rise in UHPC’s compressive strength with the rise in the proportion of NA. UHPC added with NA at the ratio of 1.0% was observed to have the highest compressive strength. When compressive strength was treated as the explained variable, UHPC performed best when the proportion of NA was 1.0%.

The compressive strength of UHPC added with NA in this work at varying ratios at the curing age of 28 days was somewhat greater than that of UHPC in the work by Li et al. [46], generally within 126.11–152.06 MPa. The promoting effect of the incorporation of NA on the cement hydration of UHPC, and the yield of hydration products [24] was the major cause of the rise in compressive strength.

### 3.4. Elastic Modulus

**Figure 6:**
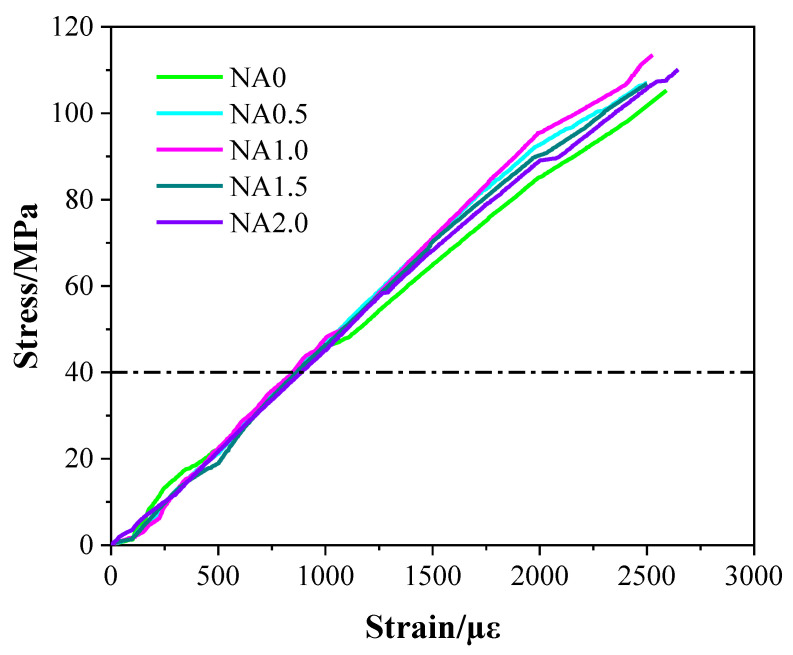
Stress-strain curves of UHPC.

**Figure 7:**
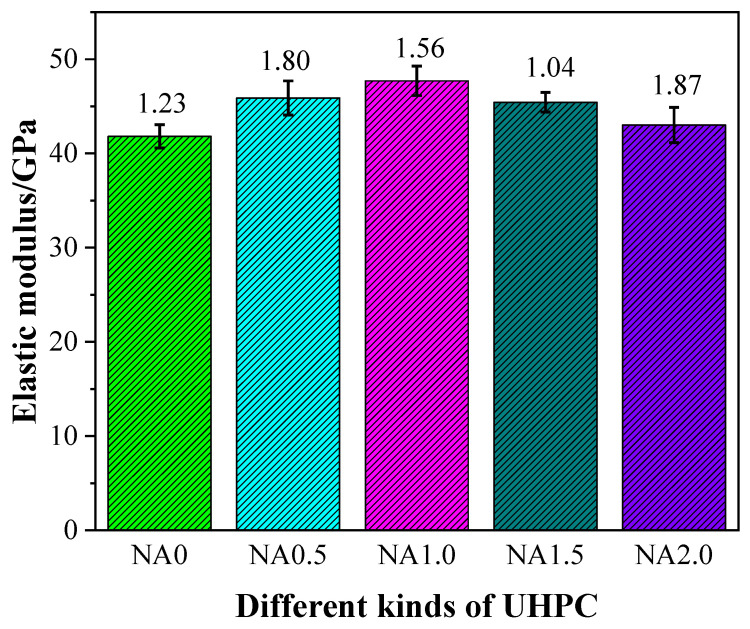
Elastic modulus of UHPC with different contents of NA.

Figure 6 displays the stress–strain curves for UHPC added with NA at varying proportions. From Figure 6, it can be seen that the rising stress of UHPC led to a linear rise in its strain, while different types of UHPC had varying slopes of stress–strain curve. In particular, NA1.0 was found to have the greatest slope of stress–strain curve. The elastic modulus of UHPC had some changes, as shown in Figure 7, due to the incorporation of NA in varying proportions. Specifically, UHPC with NA was also found to have a higher level of elastic modulus than UHPC without NA, hinting at the promoting effect of the addition of NA on the elastic modulus. The elastic moduli of the examined types of UHPC ranging from NA0 to NA2.0 were 41.82, 45.89, 47.71, 45.44, and 43.03 GPa. The elastic moduli of NA0.5, NA1.0, NA1.5, and NA2.0 were, respectively, 9.73%, 14.08%, 8.66%, and 2.89% higher than that of NA0, indicating the nonlinear changes in UHPC in elastic modulus with the rising proportion of NA. UHPC added with NA at the ratio of 1.0% was observed to have the highest elastic modulus. Therefore, when the elastic modulus was treated as the explained variable, UHPC performed best when the proportion of NA was 1.0%. The elastic modulus of UHPC added with NA in this work at varying ratios at the curing age of 28 days was somewhat greater than that of UHPC prepared with recycled fine aggregate in the literature [47], generally within 41.82–47.71 GPa. The elastic modulus and proportion of the three basic components of concrete, including the interface transition zone, cement paste, and aggregate, determine the elastic modulus of concrete [48]. The promoting effect of the addition of NA on the hydration of cement was the primary cause of the increased elastic modulus of UHPC [24].

**Figure 8:**
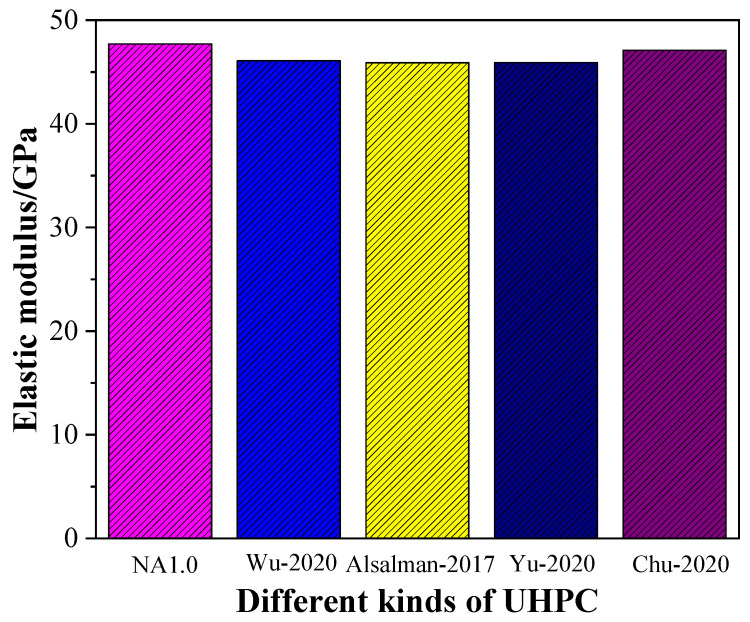
Elastic modulus of UHPC from different published papers [2,7,47,49].

Figure 8 shows the differences between different studies on UHPC in terms of the outcome of elastic modulus. As shown in Figure 8, UHPC had a water–binder ratio ranging from 0.18 to 0.22. From Figure 8, it can be observed that the UHPC made in this work had a higher elastic modulus than that of UHPC from the published papers [2,7,47,49]; thus, it could be concluded that the addition of NA into UHPC accounted for its higher elastic modulus. Compared with the previous studies on the effect of alumina micro-powder on the elastic modulus of UHPC and self-compacting mortar [1,50], the incorporation of NA led to more significant enhancement in the elastic modulus of UHPC.

From the above results, it could be seen that when the amount of NA was greater than 1.0% (the optimal amount), there were slight decreases in the three mechanical properties of UHPC. NA in a greater amount was prone to agglomeration and therefore prevented UHPC from dispersing. For this reason, the incorporation of NA in a higher-than-optimum amount led to slight decreases in the mechanical properties of UHPC.

### 3.5. Ultrasonic Pulse Velocity

**Figure 9:**
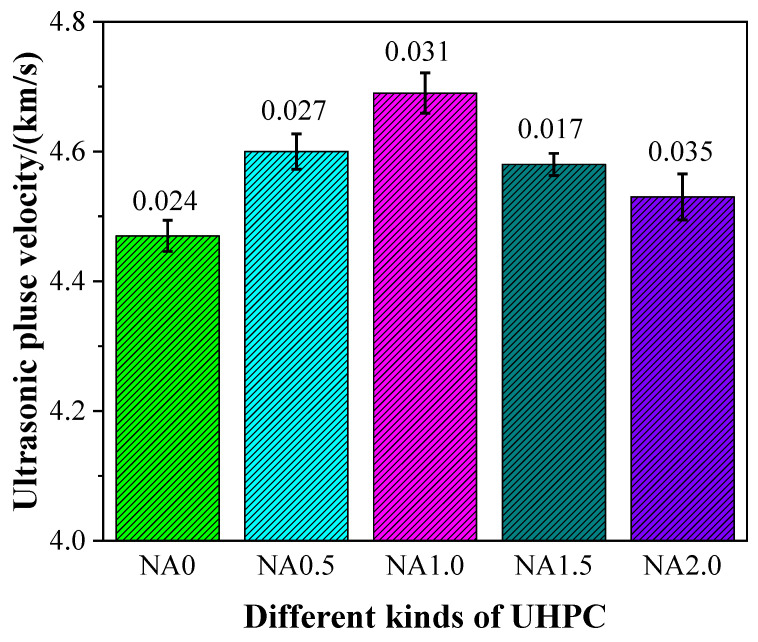
Ultrasonic pulse velocity of UHPC with different contents of NA.

The ultrasonic pulse velocity (UPV) of UHPC had some changes, as shown in Figure 9, due to the incorporation of NA at varying proportions. Specifically, UHPC with NA was found to have a higher level of UPV than UHPC without NA in all cases, hinting at the promoting effect of the addition of NA on the UPV of UHPC. The UPVs were 4.47, 4.60, 4.69, 4.58, and 4.53 km/s for the examined types of UHPC ranging from NA0 to NA2.0, indicating the nonlinear rise in UPV with the rise in the proportion of NA. UHPC added with NA at the ratio of 1.0% was observed to have the highest UPV. Therefore, when UPV was treated as the explained variable, UHPC performed best when the proportion of NA was 1.0%. The UPV of UHPC added with NA in this work at varying ratios was somewhat greater than that of reactive powder concrete that was made in a previous study with steel–polypropylene fiber [51], generally within 4.47–4.69 km/s. The UPV of UHPC was found to have a link with the compactness of its internal structure; a denser internal structure implied higher UPV and shorter time for ultrasonic pulse propagation. On the contrary, the pulse speed was lower when the internal structure was loose and the pores were increased because it took a long time to propagate the ultrasonic pulse.

### 3.6. Relationship between Compressive Strength and Elastic Modulus

**Figure 10:**
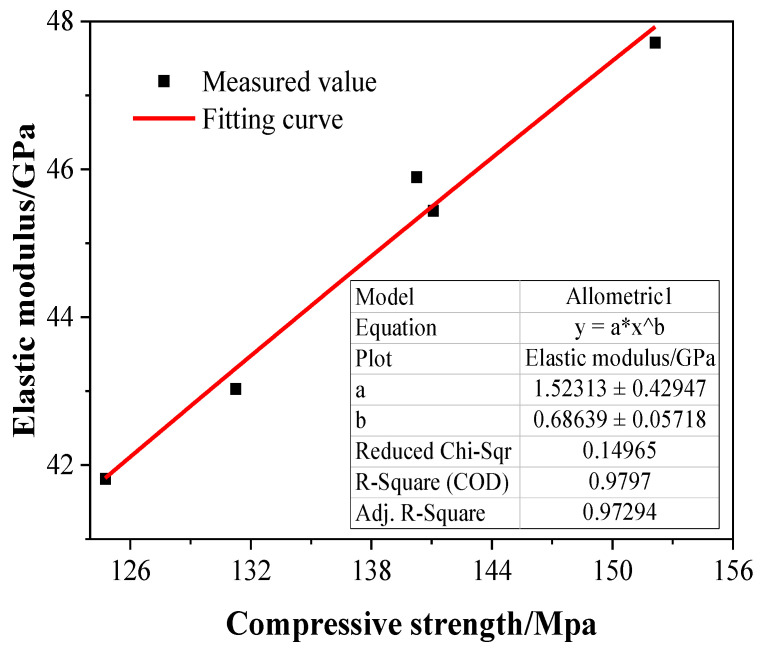
Relationship between elastic modulus and compressive strength.

Determining the elastic modulus of concrete, which requires a large test sample size, is a complicated and time-consuming process, causing some difficulties in engineering practice. Many researchers have proposed exploring how the compressive strength of concrete is correlated with its elastic modulus for this purpose, so as to calculate the elastic modulus of concrete with indirect formulas. When it comes to UHPC, relevant scholars [49,52] have also studied how its compressive strength is correlated with its elastic modulus, proposing a series of empirical formulas. The results of predicting the elastic modulus of UHPC by following these empirical formulas using present test data were shown in Figure 10. The following Equation (4) was the equation of their relationship:

(4) $$E_{C}=1.523f_{cu}^{0.686}$$

$E_{c}$ is elastic modulus, while $f_{cu}$ stands for compressive strength.

The R-square from Equation (4) was 0.9797, consistent with the empirical result obtained in the work of Jurowski et al. [53]. It can be inferred that compressive strength could be an ideal option for predicting elastic modulus via Equation (4).

### 3.7. Drying Shrinkage

**Figure 11:**
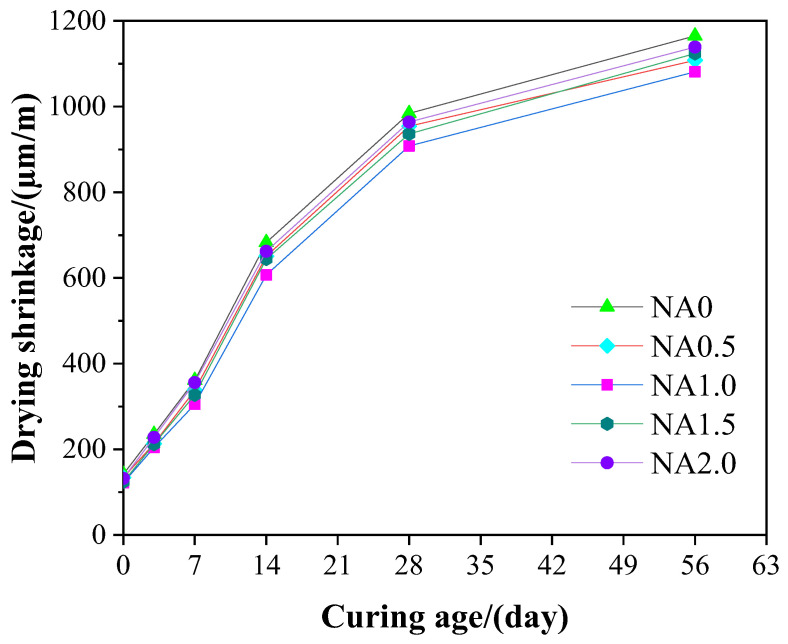
Drying shrinkage of UHPC at different curing ages.

Figure 11 shows how the drying shrinkage of UHPC at different curing ages changed under the action of different contents of NA. Specifically, the drying shrinkage of UHPC rose rapidly and then stabilized when the curing days increased. UHPC in this work shared a similar pattern with self-compacting mortar in the literature [54] in terms of the drying shrinkage variation. At each curing age, the incorporation of NA into UHPC resulted in a decrease in drying shrinkage. The increase in NA in UHPC had a U-shaped relationship with drying shrinkage. When the NA content was 1.0%, UHPC was found to have the smallest drying shrinkage. From the perspective of drying shrinkage, UHPC was deemed to perform best when added with NA at a ratio of 1.0%. At the curing age of 28 days, NA0.5, NA1.0, NA1.5, and NA2.0 had decreases of 4.41%, 11.76%, 5.88%, and 2.90%, respectively, compared with NA0. The addition of NA in UHPC had a prohibiting effect on drying shrinkage, a promoting effect on the durability, and a prohibiting on cracking risk. The change tendency of UHPC in mechanical properties and UPV was also followed by its drying shrinkage. Basically, UHPC with better mechanical properties was found to have lower drying shrinkage, consistent with the measures presented in the work of Ledesma et al. [55].

### 3.8. Porosity and Pore Size Distribution

**Figure 12:**
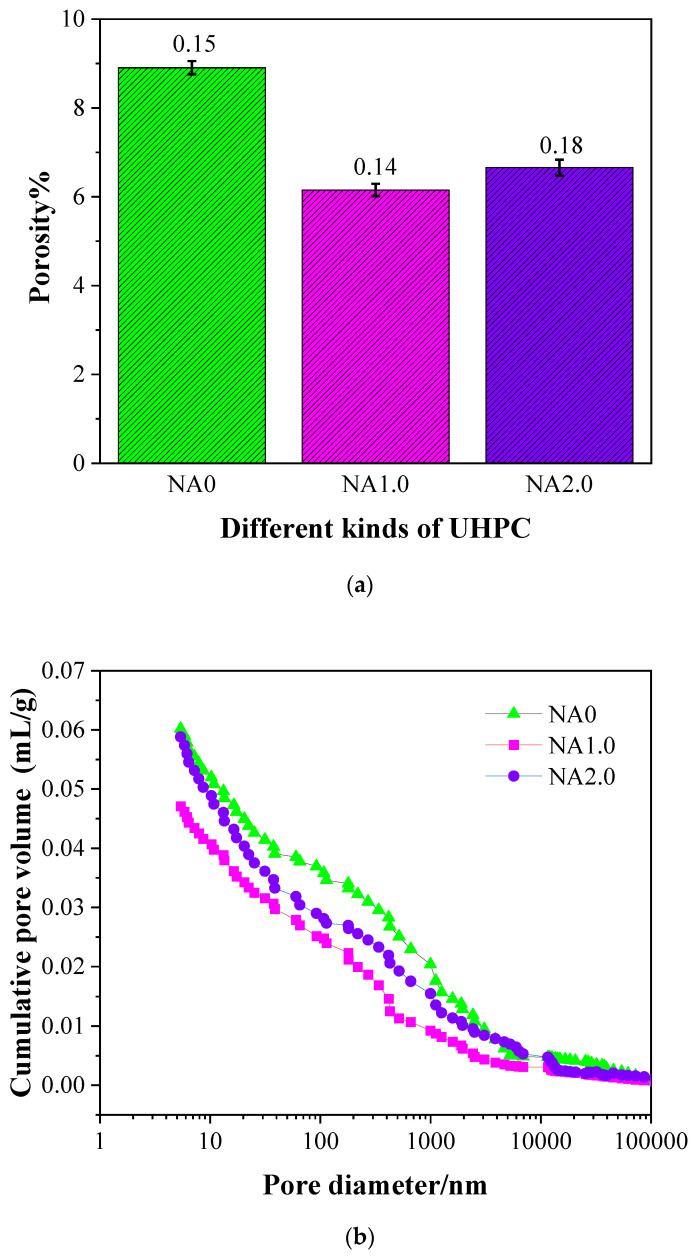

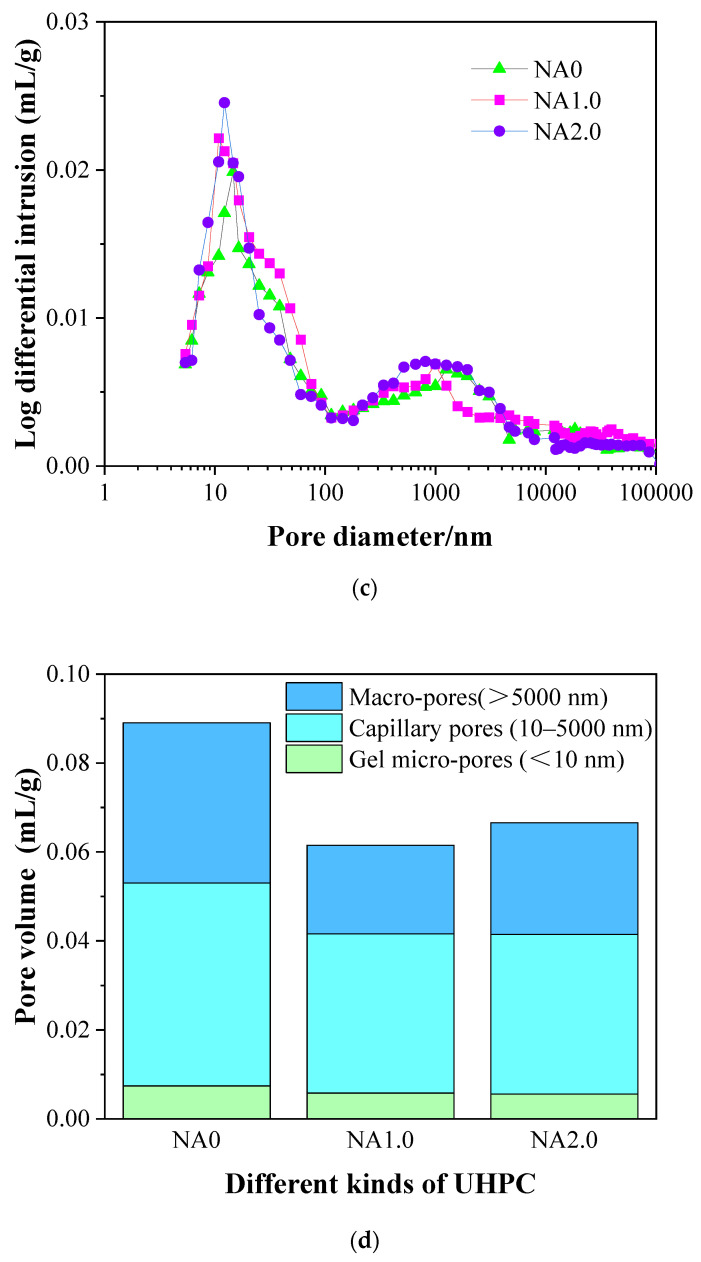
Porosity and pore structure of different kinds of UHPC: (**a**) Porosity; (**b**) Cumulative pore volume; (**c**) Pore size distribution; (**d**) Pore volume fraction.

The changes in concrete properties rely on its pore size distribution and porosity to a large extent [56]. The changes in UHPC porosity with the changes in the amount of NA (i.e., 0%, 1.0%, and 2.0%) are presented in Figure 12a. According to Figure 12a, the porosity of UHPC with NA was never higher than that of its NA-free counterpart, hinting at the prohibiting effect of NA on the porosity of UHPC. A tendency of declining followed by rising could be observed from the porosity of NA0, NA1.0, and NA2.0, which were 8.91%, 6.15%, and 6.65%, respectively. This tendency was the evidence of the nonlinear relationship between NA and porosity. UHPC added with NA at the ratio of 1.0% was found to have the smallest porosity. When porosity was treated as the explained variable, UHPC performed best when the proportion of NA was 1.0%.

The changes in UHPC in cumulative pore volume with the changes in the amount of NA are presented in Figure 12b. Accordingly, the addition of NA in UHPC was observed to have a prohibiting effect on its cumulative pore volume, hinting at the feasibility of incorporating NA into UHPC at a proper proportion to optimize its pore structure.

The changes of UHPC in pore size distribution with the changes in the amount of NA are presented in Figure 12c. The amount of NA in UHPC was found to have no impact on the law of pore size distribution, each with a typical peak that corresponded to the threshold pore diameter of each type of UHPC. Specifically, the threshold pore diameter of NA0, NA1.0, and NA2.0 was 14.62 nm, 10.81 nm, and 12.19 nm, respectively, which also showed the trend of decreasing followed by rising, which was consistent with the change tendency in the aspects of porosity. The experimental results herein were consistent with the threshold pore diameter obtained by Soliman et al. [57] at a level around 10 nm. Compared with NA0, the threshold pore diameter of NA1.0 and NA2.0 were lower, which indicated that NA could improve the pore structure of UHPC.

From the perspective of size, concrete is broadly believed to contain three types of pores, namely gel pores (<10 nm), capillary pores (10–5000 nm), and macropores (>5000 nm) [58]. Figure 12d presents the distribution of different pores in UHPC added with NA at varying proportions. Table 3 lists the proportions of different pores in UHPC. According to what could be observed from Figure 12d and Table 3, the ratios of capillary pores to total pores were 51.19%, 58.11%, and 53.81%, respectively, for NA0, NA1.0, and NA2.0, reflecting a negative relationship with porosity and a reversed U-shape changing trend. This hinted at the promoting effect of NA on the pore structure of UHPC.

This promoting effect of NA on pore structure and the prohibiting effect on porosity might be attributed to the following reasons: First, NA had a promoting effect on the yield of hydration products in UHPC, its hydration degree, and internal pore structure and had a prohibiting impact on its porosity [24]; meanwhile, the particles of NA in the size of 10 nm, as reflected in Figure 2, filled the internal pores of UHPC and therefore reduced the porosity and improved the pore structure; in addition, hydrated calcium aluminate generated from the NA-Ca(OH) _2_ chemical reaction gave rise to another compact network structure [25].

### 3.9. Microstructure

**Figure 13:**
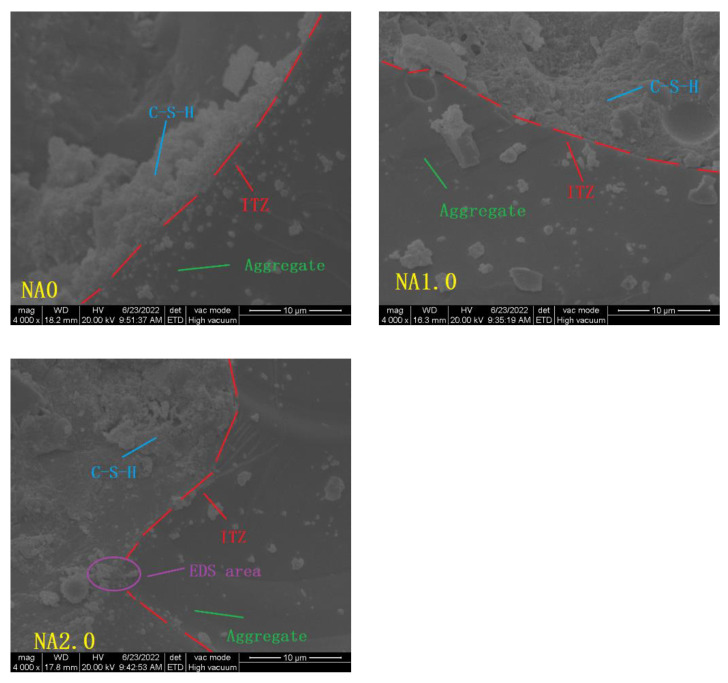
Micrographs of different kinds of UHPC at curing age of 28 days.

Figure 13 shows the changes of UHPC in micromorphology with the changes of the amount of NA. From Figure 13, it can be seen that different types of UHPC had a relatively compact microstructure, in which micro holes and cracks were absent, which corresponded to the low porosity of UHPC. Although the NA0 microstructure had no obvious cracks, it did not perform well in the uniformity and compactness of its interface transition zone (ITZ). NA1.0 had a more uniform and compact ITZ than NA0, and no obvious interface relationship could be seen.

**Figure 14:**
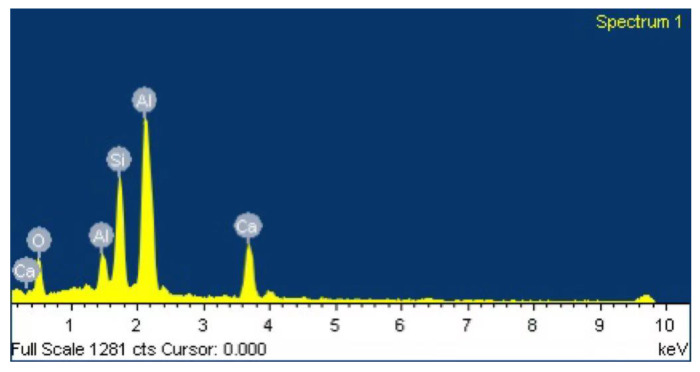
EDS results of the selected zones in Figure 13.

From the results of EDS in Figure 14 and Table 4, it can be seen that unhydrated NA existed in a large amount in NA2.0, agglomerating and adhering to the surface, so that its microstructure became less compact and uniform, corresponding to the relatively high porosity of UHPC. NA had a small particle size. On the one hand, it could fill the internal pores of UHPC. On the other hand, the cement hydration gave rise to the Ca(OH)_2_ reaction. Chemical bonding accelerated the generation of C-A-S-H, C-A-H, and C-S-H. In the process, Ca(OH)_2_ reacted to form a new dense structure [25].

To sum up, compared with UHPC without NA, the UHPC containing NA was found to have higher slump flow, higher compressive strength, higher flexural strength, higher elastic modulus, higher UPV, lower porosity, and lower drying shrinkage. NA could improve the fluidity, microstructure, and pore structure of UHPC. The mechanical properties and the durability of UHPC improved because of the incorporation of NA. The optimal amount of NA in UHPC was higher than that of nano-SiO_2_, graphene oxide, nano-CaCO_3_, and nano-TiO_2_ (as indicated in reference [2,11,13,14,15]). On the whole, the mechanical performance improvement of UHPC in this work via NA was similar to that of the other kinds of concretes modified by the other nanomaterials (as indicated in reference [2,13,20]).

## 4. Conclusions

As a kind of supplementary cementitious material, NA was used in this study under the MAA particle-packing model to seek the best content of NA that maximized the parameters of UHPC. Systematic examination was conducted of the changes of UHPC in durability, mechanical properties, and workability, as well as the changes in UPV and microstructure, under the actions of NA at varying ratios. According to the results of the research, the following main conclusions could be obtained:

1. The UHPC specimens made herein had a slump flow ranging from 235 to 255 mm. The incorporation of NA in a proper amount could improve the fluidity of UHPC.
2. The flexural strength, compressive strength, and elastic modulus of UHPC were 22.84–24.86 MPa, 131.25–152.06 MPa, and 43.03–47.71 GPa, respectively. The mechanical properties of UHPC could be improved by NA.
3. The UPV propagated in UHPC ranged from 4.47 to 4.69 km/s. The addition of NA led to an increase of 1.3%-4.9% in the UPV of UHPC.
4. The porosity of UHPC was between 6.15% and 8.91%. The addition of NA had a prohibiting impact on the threshold pore diameter and porosity of UHPC.
5. The incorporation of NA led to a decline of 2.9–11.76% in the dry shrinkage of UHPC.
6. The optimal amount of NA in UHPC was 1.0% whether the explained variable used to examine NA was workability, mechanical properties, microstructure, or durability.

## Figures and Tables

**Table 1:** Chemical composition of cement, fly ash, silica fume, and quartz sand (wt.%).

Chemical Composition	Cement	Fly Ash	Silica Fume	Quartz Sand
CaO	56.214	4.656	0.895	0.036
SiO_2_	23.839	54.708	97.515	96.469
Al_2_O_3_	8.155	35.081	0.736	2.520
Fe_2_O_3_	3.600	4.429	0.188	0.316
MgO	4.252	0.235	0.238	0.235
SO_3_	3.317	0.65	0.057	-
K_2_O	0.446	0.112	0.213	0.363
Na_2_O	0.177	0.129	0.158	0.061
Specific gravity (kg/m^3^)	3050	2300	2150	2650
Specific surface (m^2^/kg)	434.2	283.9	1324	-

**Table 2:** Mix proportions of UHPC (kg/m^3^).

Mixture	NA0	NA0.5	NA1.0	NA1.5	NA2.0
Cement	649.65	646.4	643.15	639.90	636.66
Fly ash	299	299	299	299	299
Silica fume	120.4	120.4	120.4	120.4	120.4
NA	0	3.25	6.5	9.75	12.99
Quartz sand	1062.65	1062.65	1062.65	1062.65	1062.65
Water	200.0	200.0	200.0	200.0	200.0
Steel fiber	156.8	156.8	156.8	156.8	156.8
Water-reducing agent	10.69	10.69	10.69	10.69	10.69

**Table 3:** Specific contents of macro-pores, capillary pores, and gel micro-pores in UHPC with different contents of NA (%).

Mixture	NA0	NA1.0	NA2.0
Macro-pores	40.48	32.43	37.76
Capillary pores	51.19	58.11	53.81
Gel micro-pores	8.33	9.46	8.43

**Table 4:** EDS area analysis results.

Element	Weight %	Atomic %
O K	51.29	63.98
Al K	48.71	36.02

## Data Availability

The data presented in this study are available on request from the corresponding author.
